# Supplementary figures and images for: STAMBPL1 activates the GRHL3/HIF1A/VEGFA axis through interaction with FOXO1 to promote angiogenesis in triple-negative breast cancer
Source: eLife. 2025 Apr 10;13:RP102433. doi: 10.7554/eLife.102433 (PMC11984952; doi:10.7554/eLife.102433)

Figure 1A:

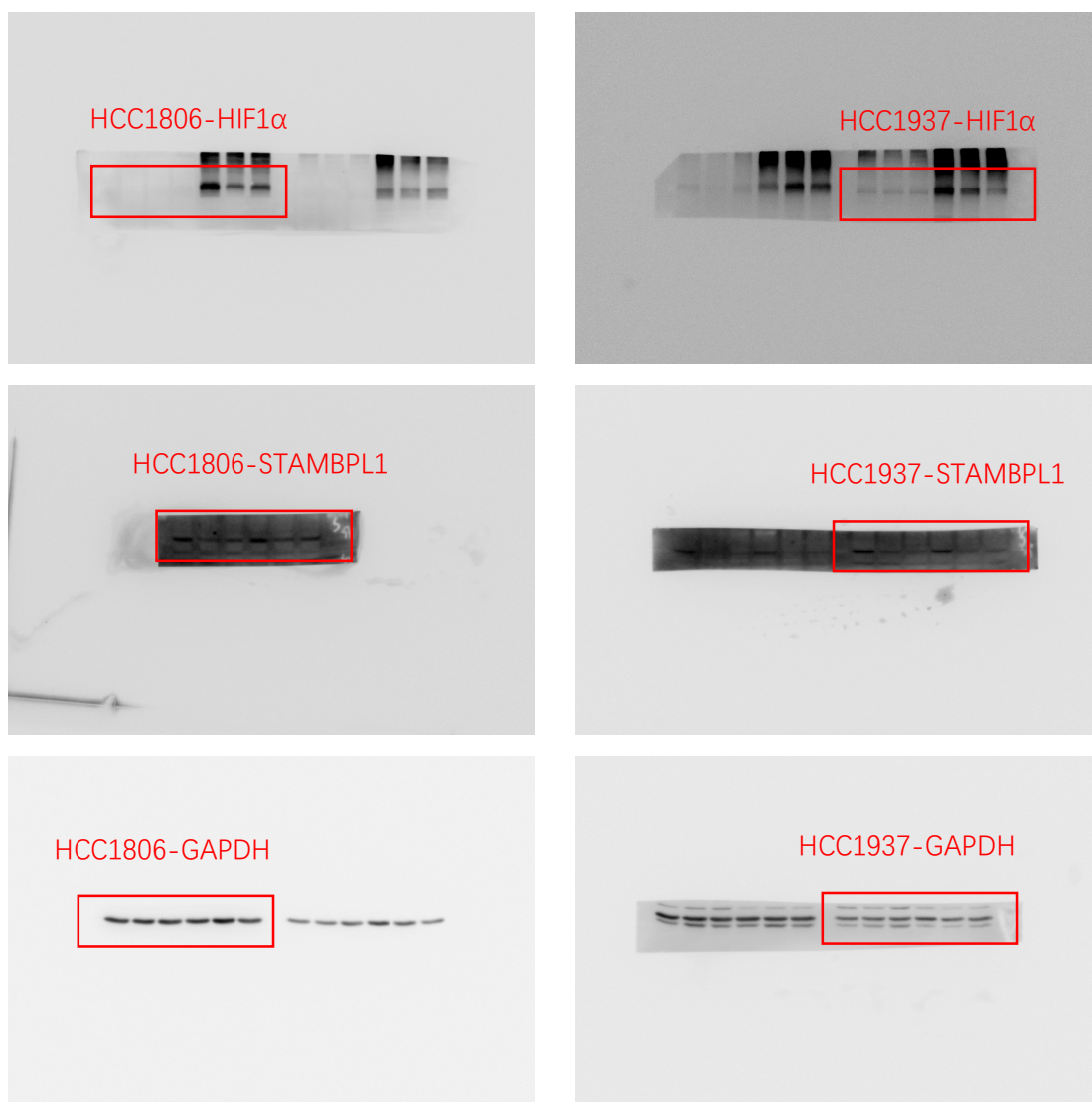

Figure 1F:

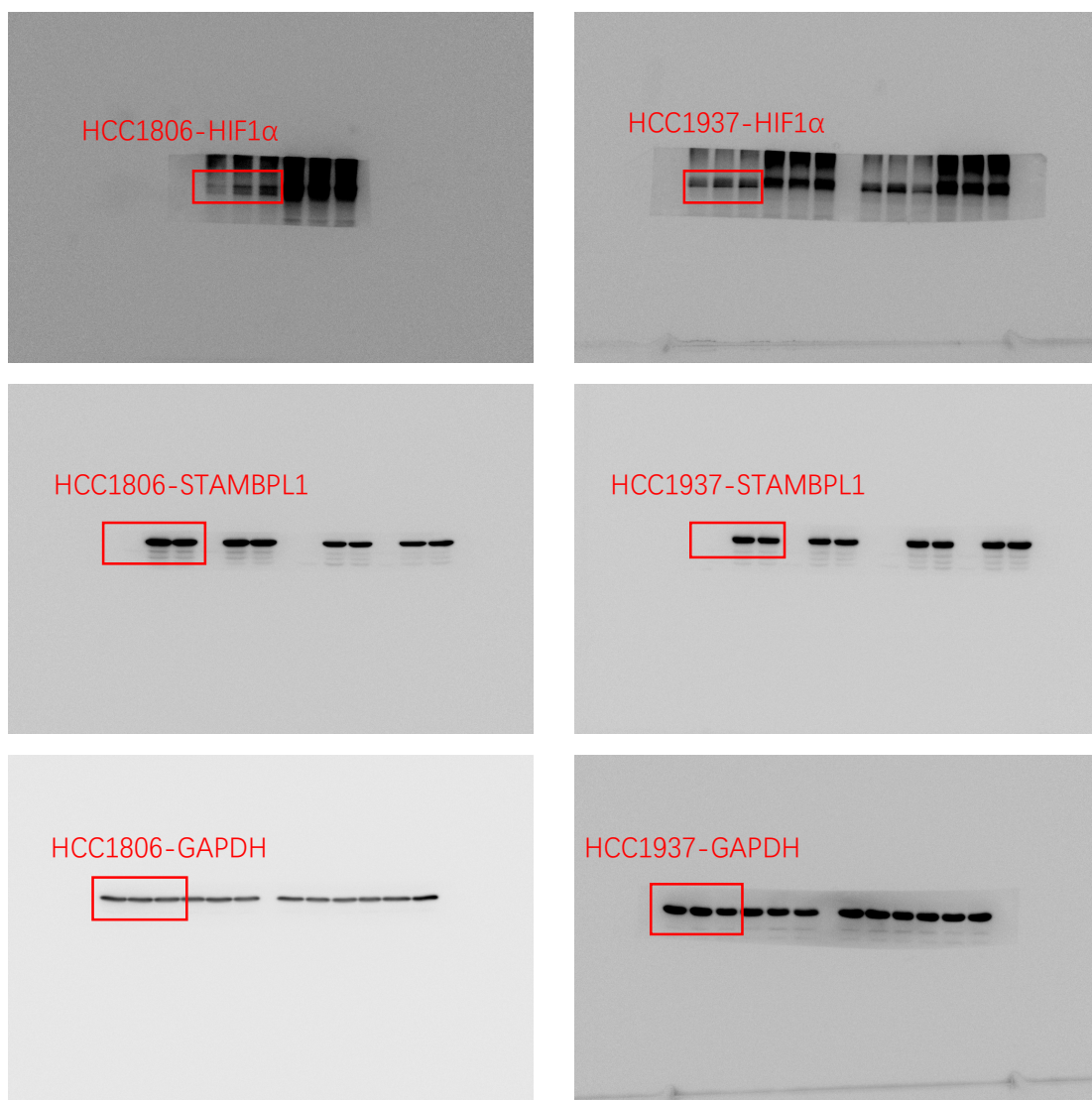

Supplement: Figure 1—source data 1. [file elife-102433-fig1-data1.zip › Figure 1-source data 1/Figure 1-source data 1.pdf]

Figure 2A:

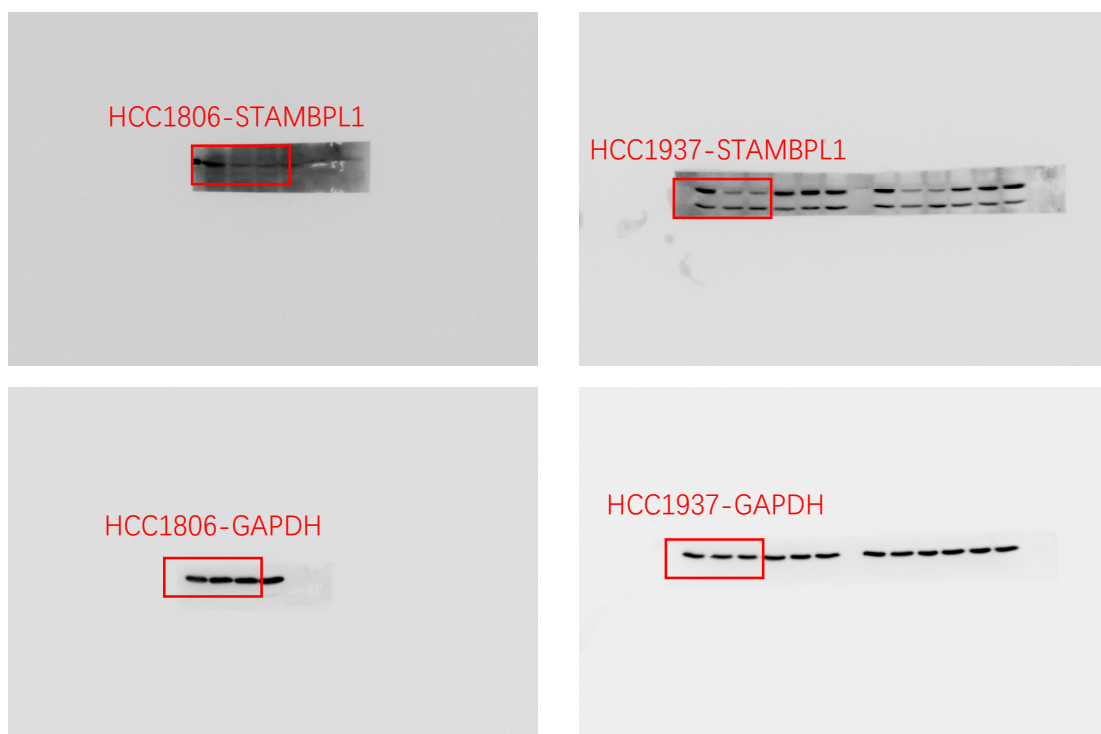

Figure 2D:

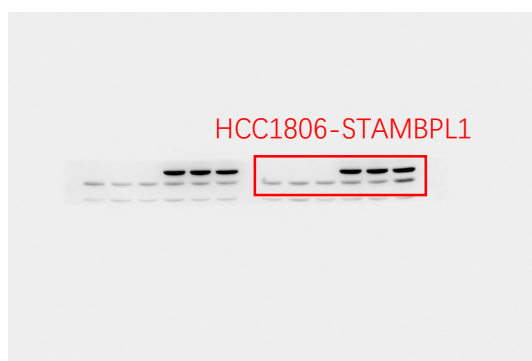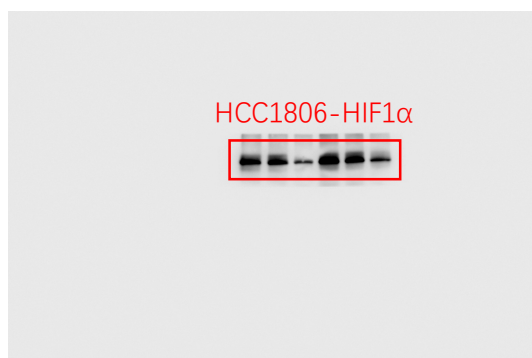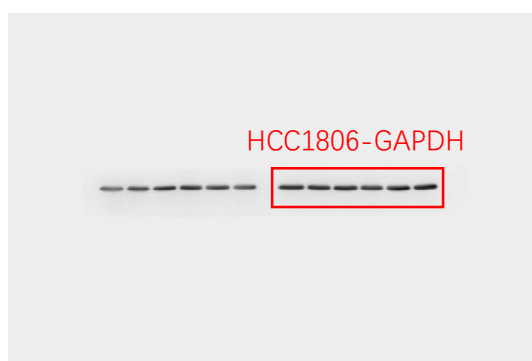

Figure 2F:

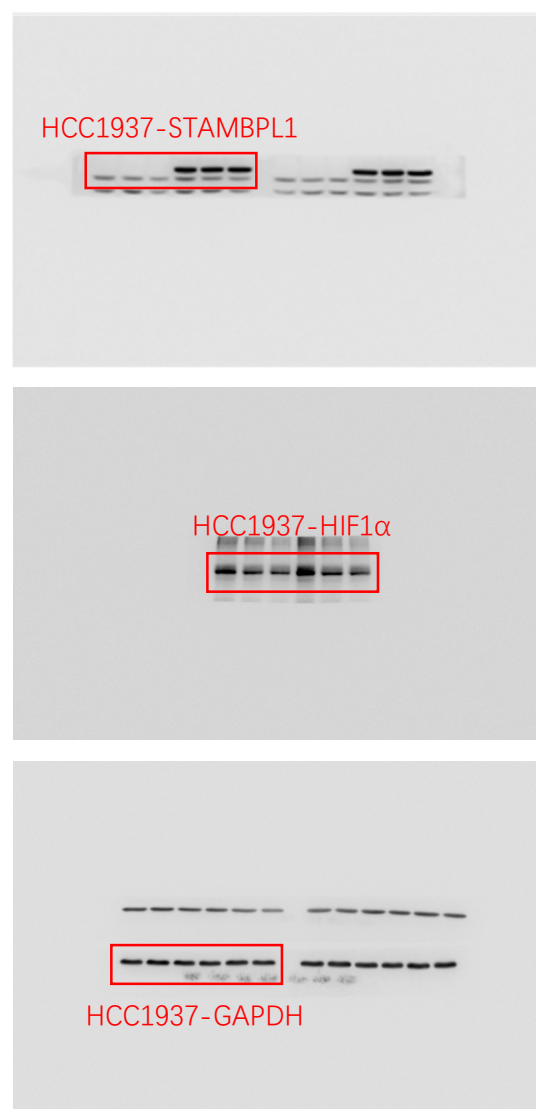

Supplement: Figure 2—source data 1. [file elife-102433-fig2-data1.zip › Figure 2-source data 1/Figure 2-source data 1.pdf]

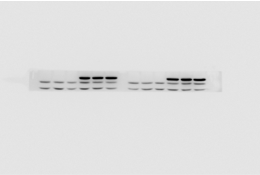

Supplement: Figure 2—source data 2. [file elife-102433-fig2-data2.zip › Figure 2-source data 2/Fig2F-HCC1937-STAMBPL1.tif]

Figure 4C:

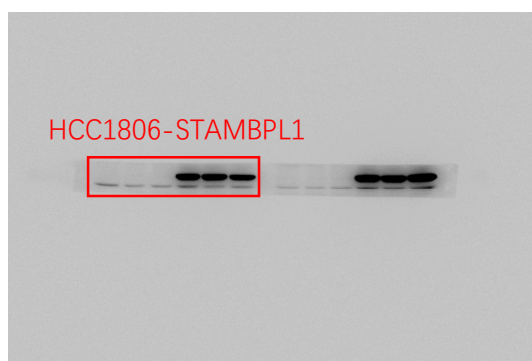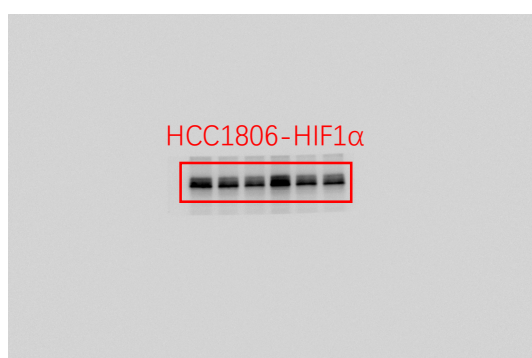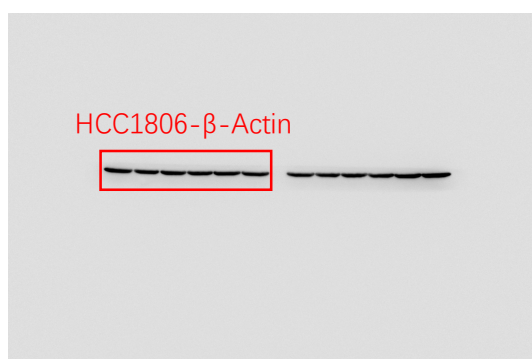

Supplement: Figure 4—source data 1. [file elife-102433-fig4-data1.zip › Figure 4-source data 1/Figure 4-source data 1.pdf]

Figure 4-figure supplement 1C:

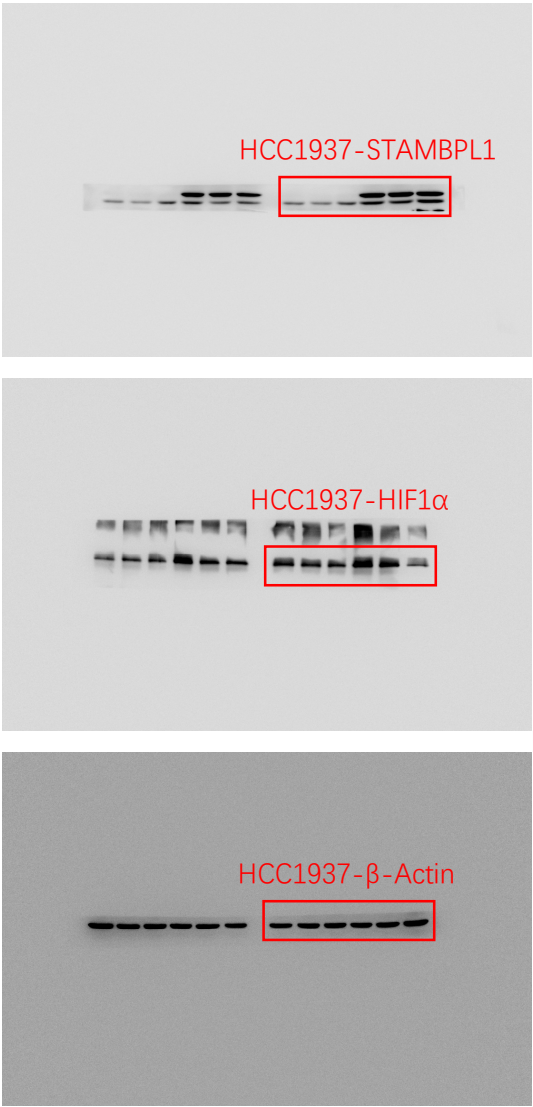

Supplement: Figure 4—figure supplement 1—source data 1. [file elife-102433-fig4-figsupp1-data1.zip › Figure 4-figure supplement 1-source data 1/Figure 4-figure supplement 1-source data 1.pdf]

Figure 5C:

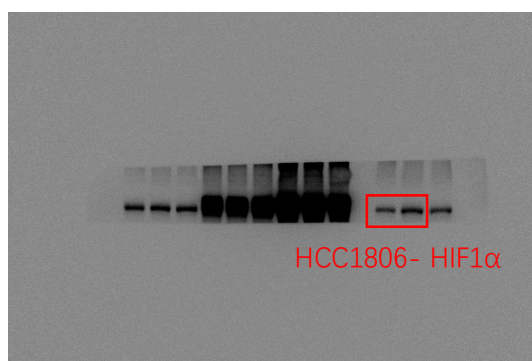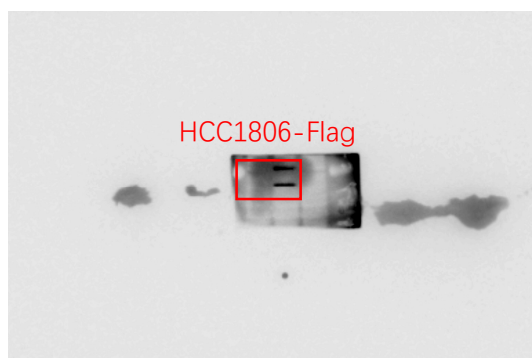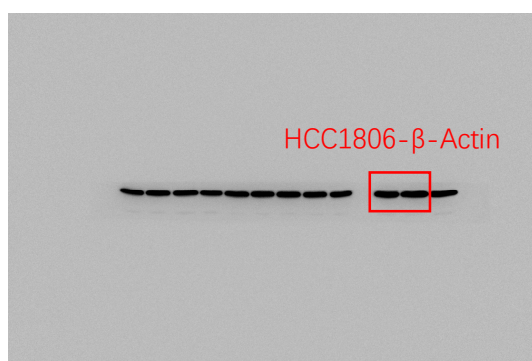

Figure 5D:

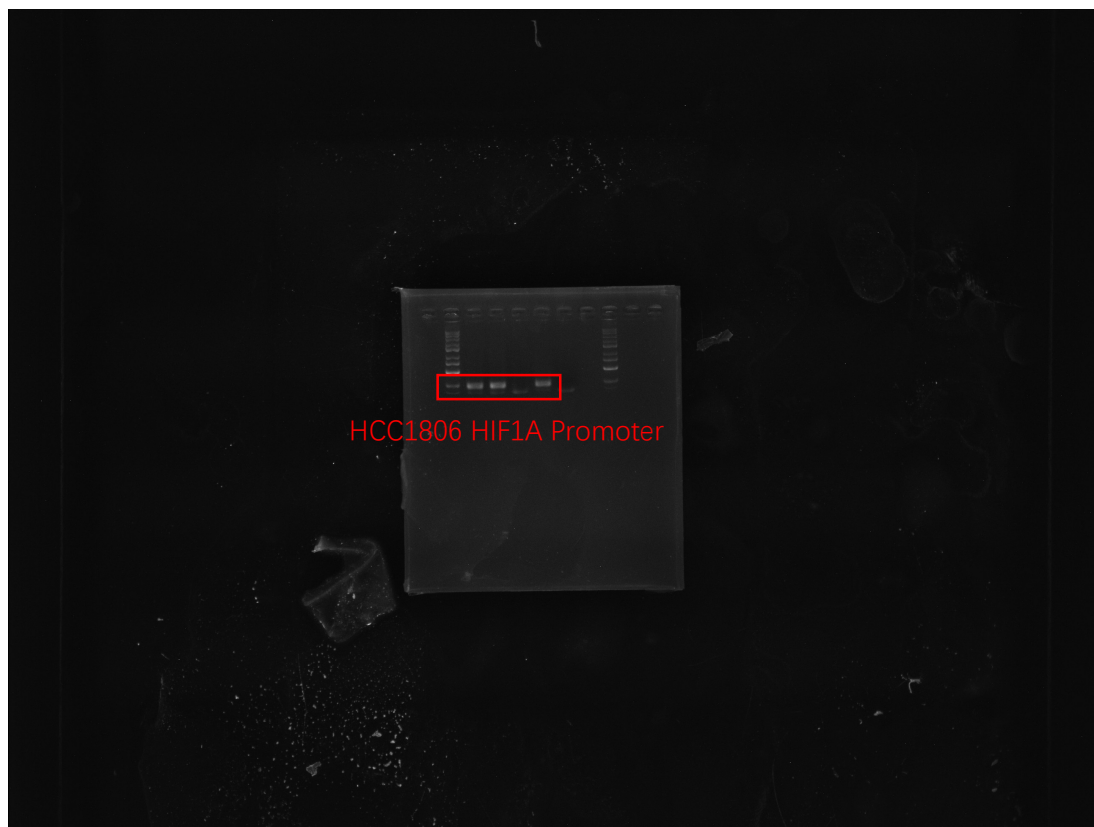

Figure 5F:

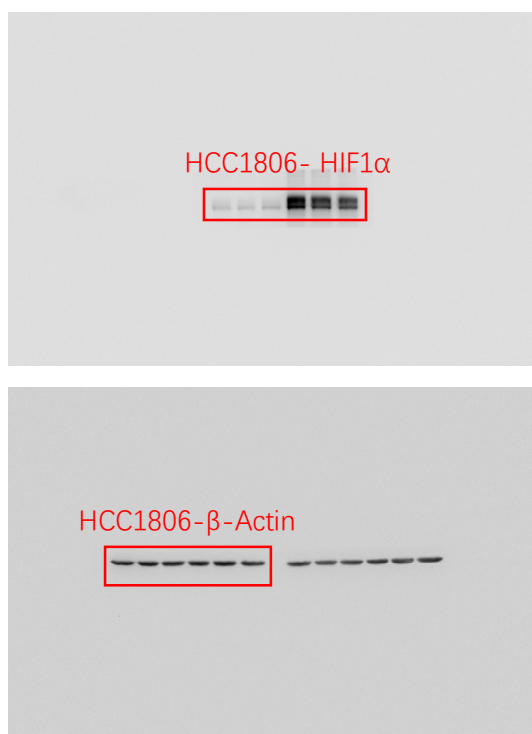

Supplement: Figure 5—source data 1. [file elife-102433-fig5-data1.zip › Figure 5-source data 1/Figure 5-source data 1.pdf]

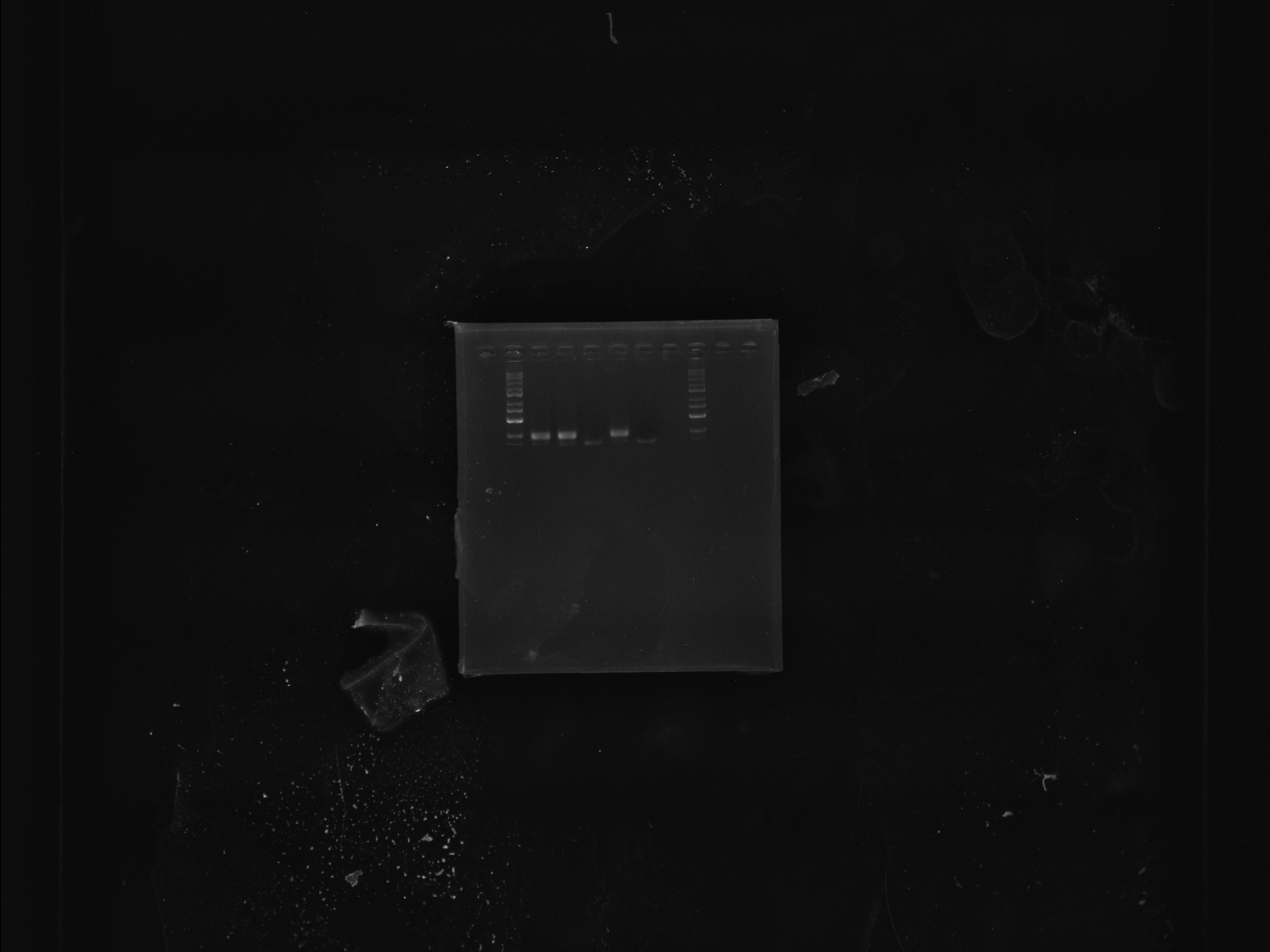

Supplement: Figure 5—source data 2. [file elife-102433-fig5-data2.zip › Figure 5-source data 2/Fig5D-HCC1806-ChIP-HIF1A promoter.tif]

Figure 5-figure supplement 1A:

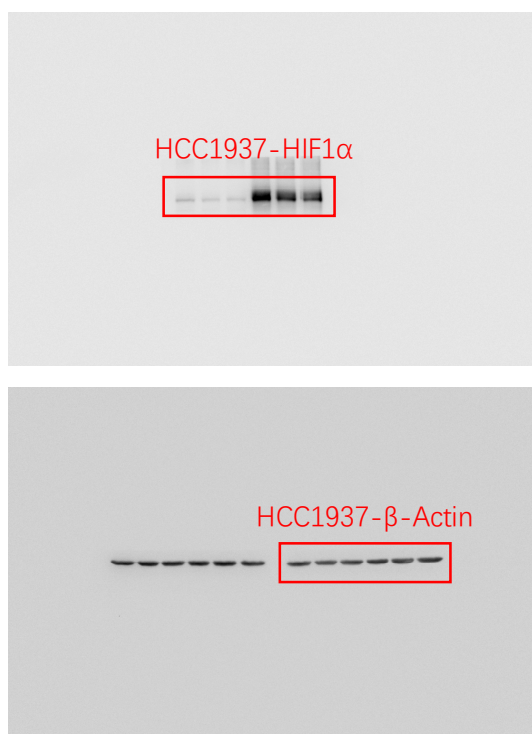

Supplement: Figure 5—figure supplement 1—source data 1. [file elife-102433-fig5-figsupp1-data1.zip › Figure 5-figure supplement 1-source data 1/Figure 5-figure supplement 1-source data 1.pdf]

Figure 6A:

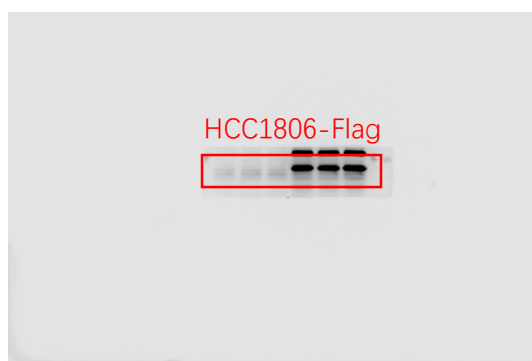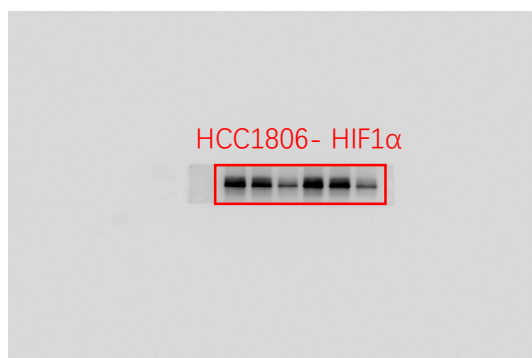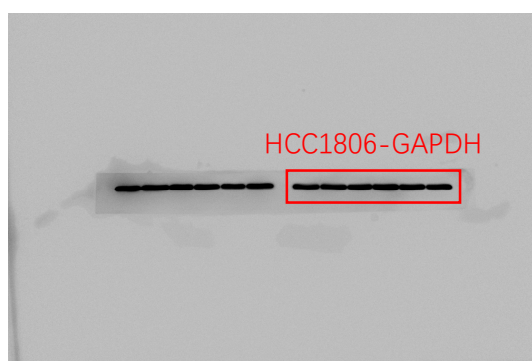

Supplement: Figure 6—source data 1. [file elife-102433-fig6-data1.zip › Figure 6-source data 1/Figure 6-source data 1.pdf]

Figure 6-figure supplement 1A:

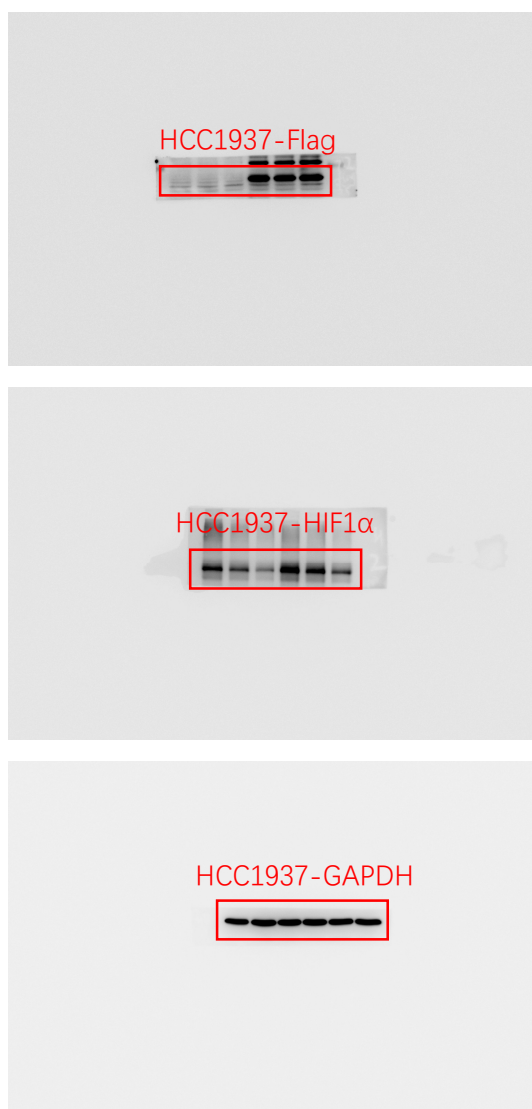

Supplement: Figure 6—figure supplement 1—source data 1. [file elife-102433-fig6-figsupp1-data1.zip › Figure 6-figure supplement 1-source data 1/Figure 6-figure supplement 1-source data 1.pdf]

Figure 7A:

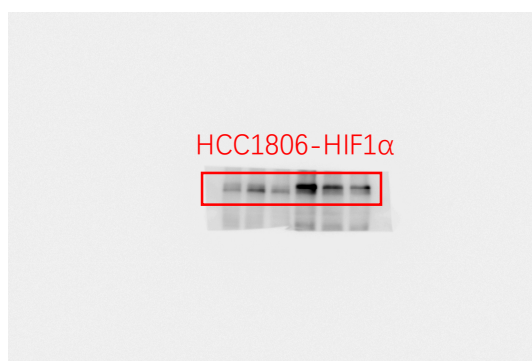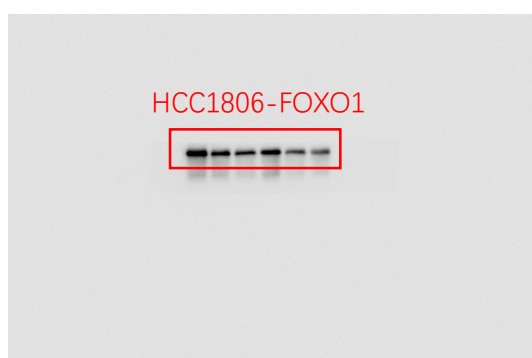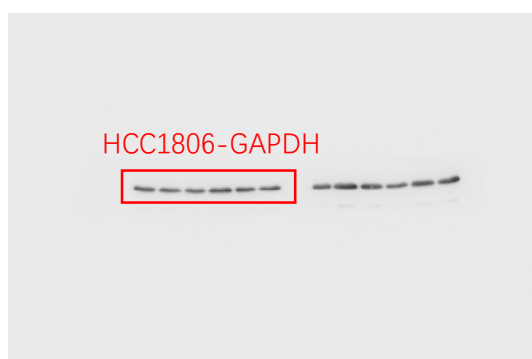

Figure 7E:

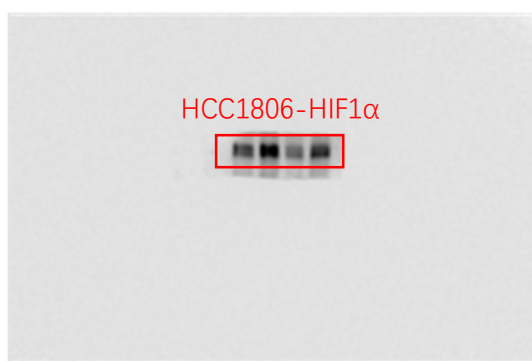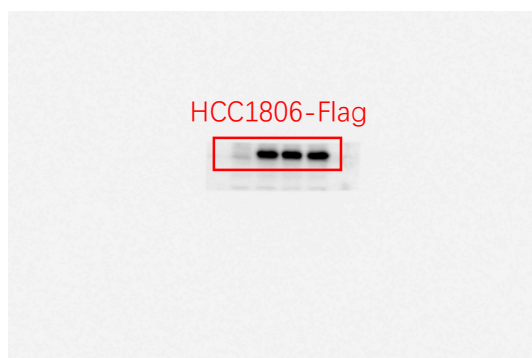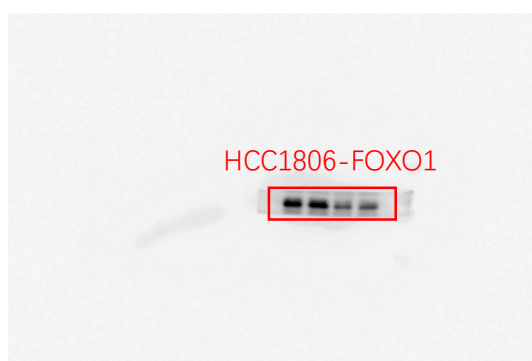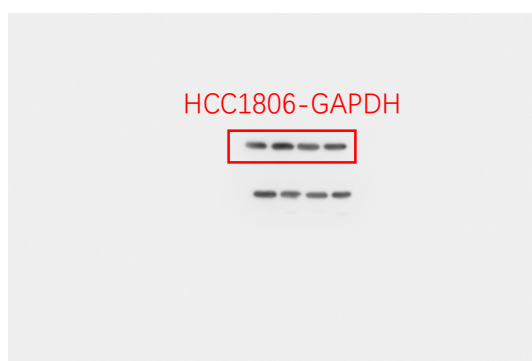

Figure 7I:

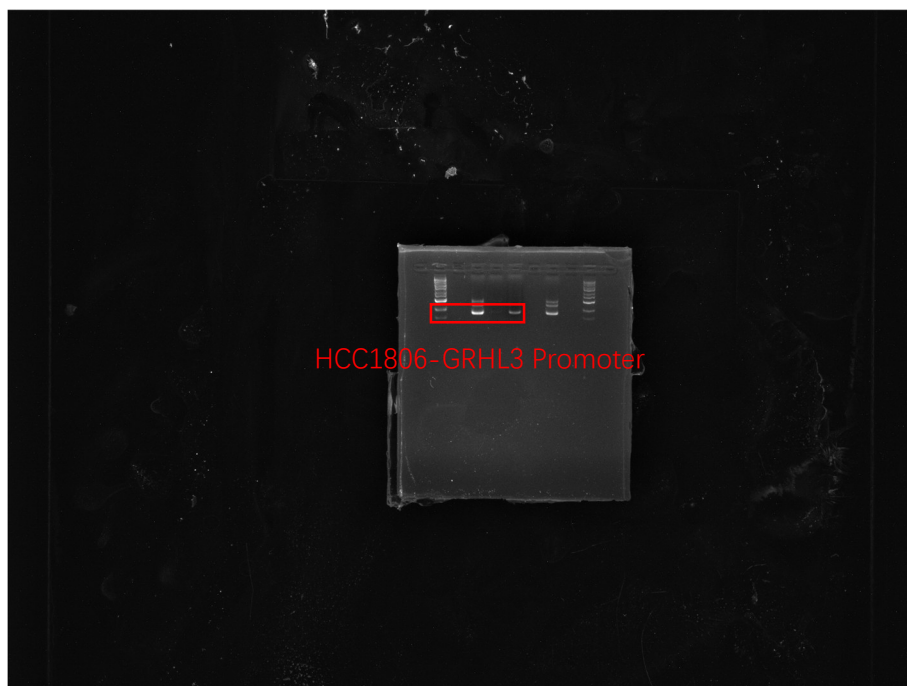

Figure 7L:

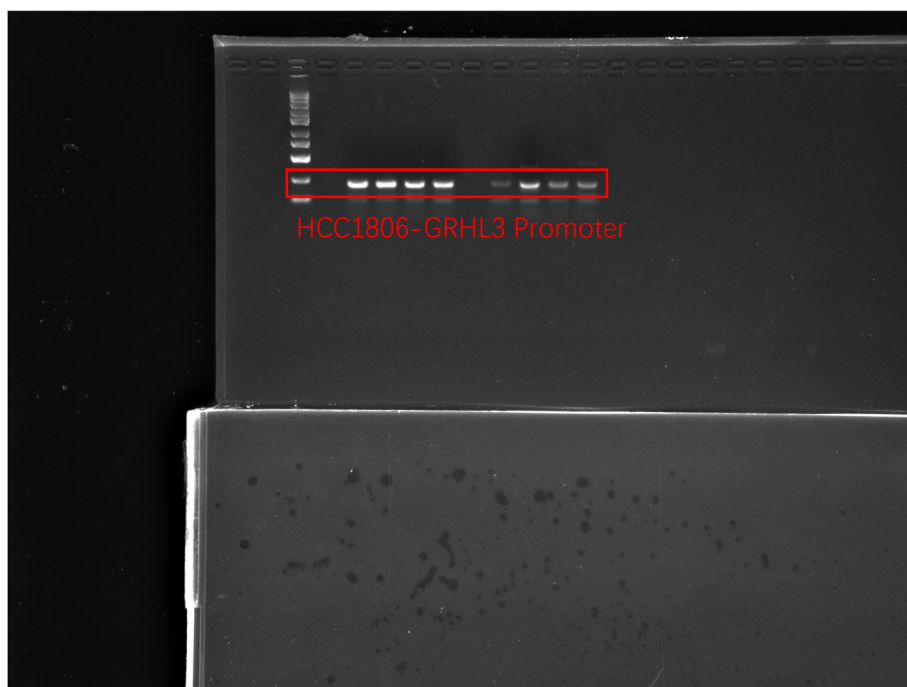

**Figure 7N:**

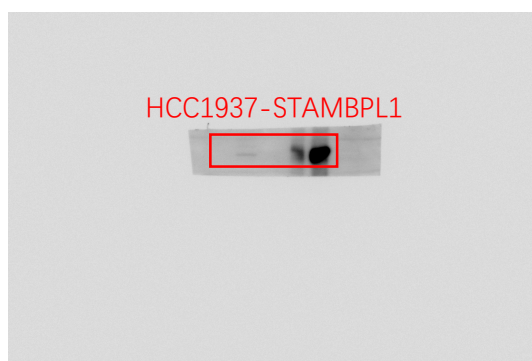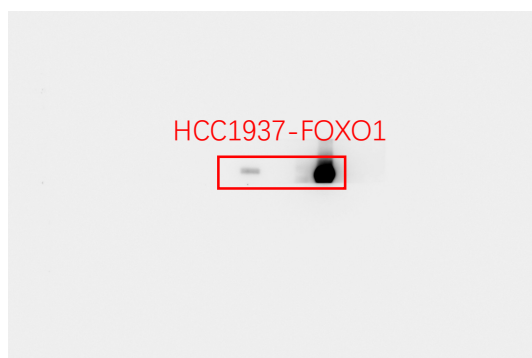

Supplement: Figure 7—source data 1. [file elife-102433-fig7-data1.zip › Figure 7-source data 1/Figure 7-source data 1.pdf]

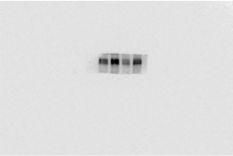

Supplement: Figure 7—source data 2. [file elife-102433-fig7-data2.zip › Figure 7-source data 2/Fig7E-HCC1806-HIF1A.tif]

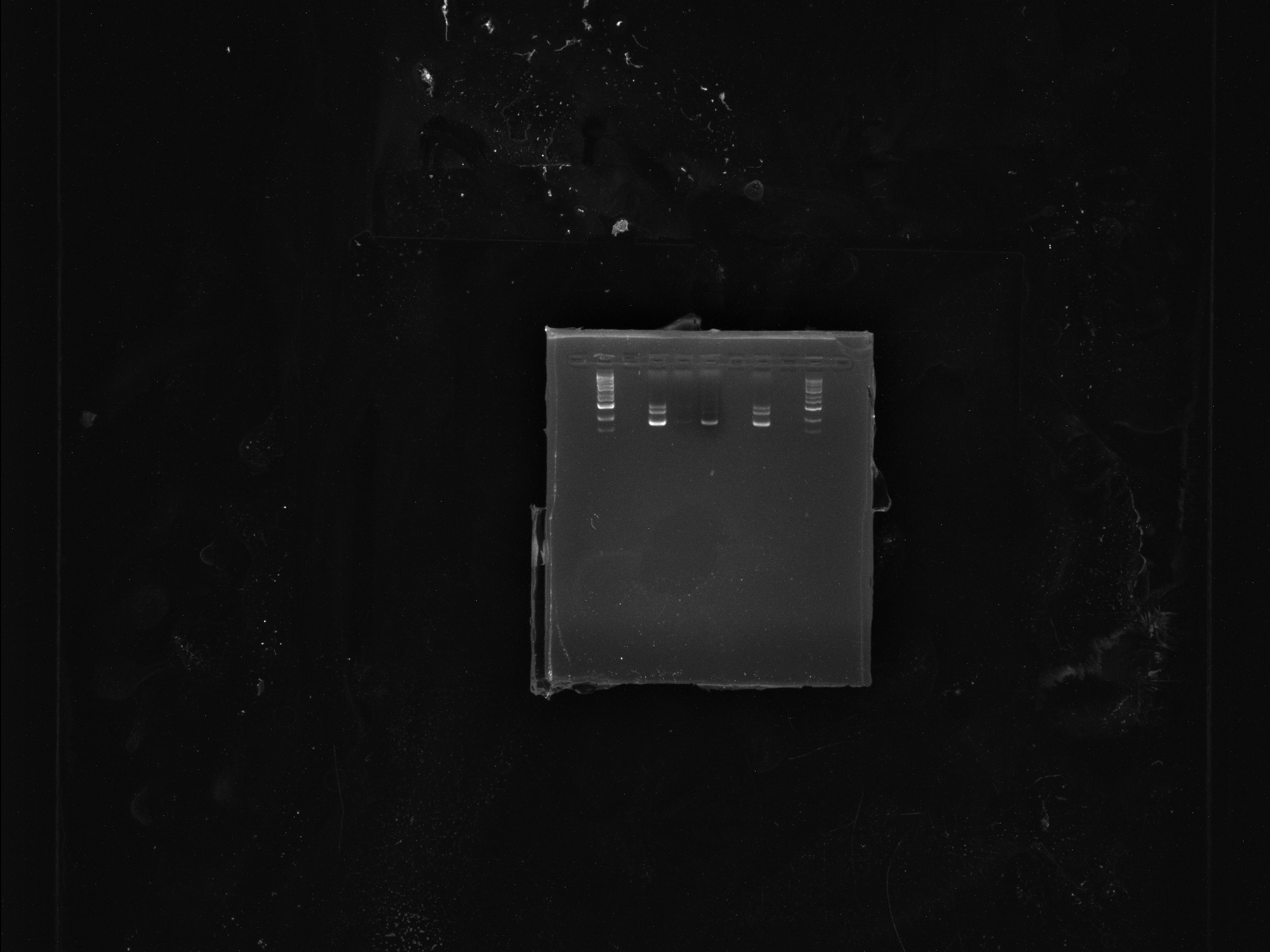

Supplement: Figure 7—source data 2. [file elife-102433-fig7-data2.zip › Figure 7-source data 2/Fig7I.tif]

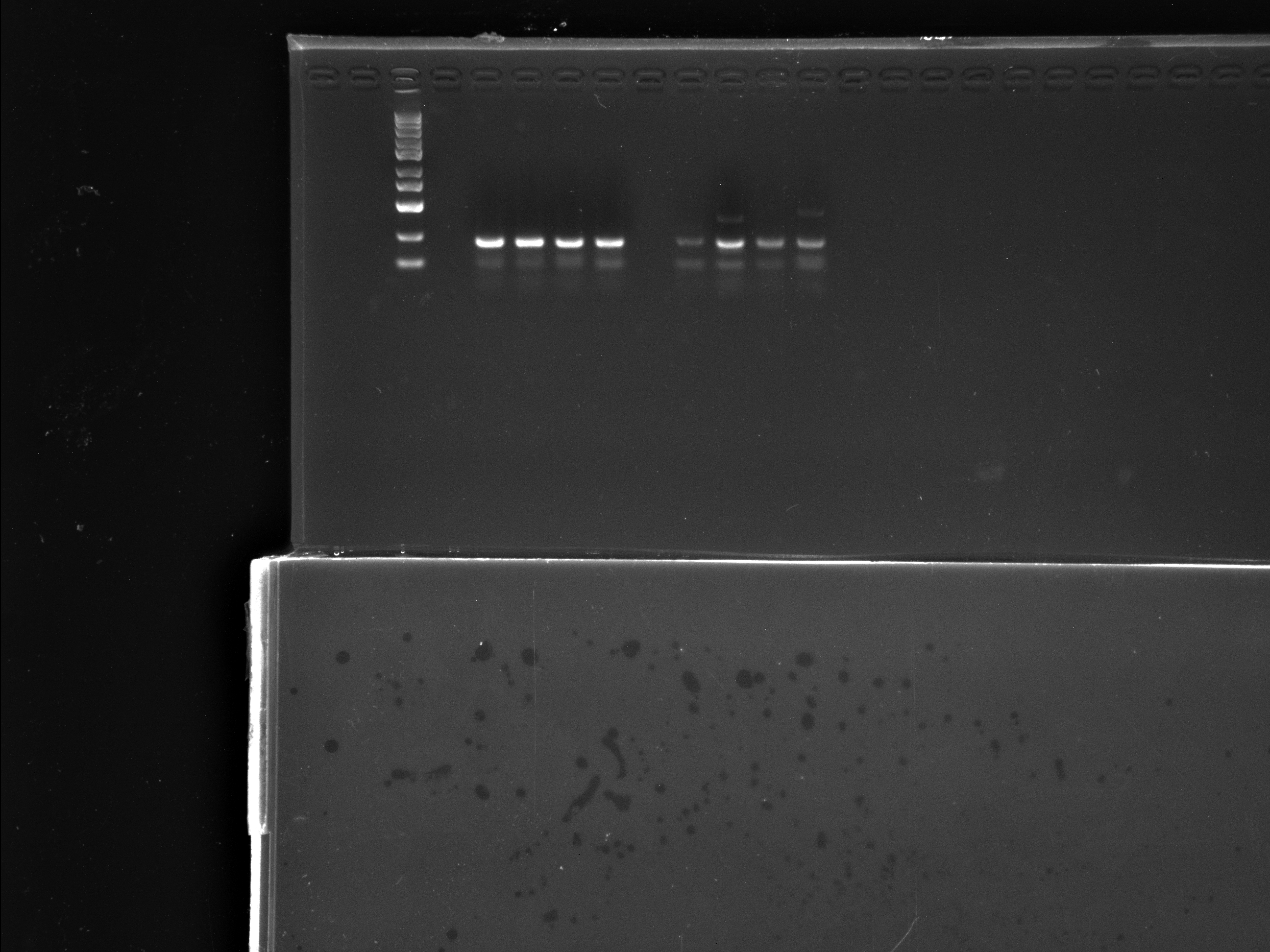

Supplement: Figure 7—source data 2. [file elife-102433-fig7-data2.zip › Figure 7-source data 2/Fig7L.tif]

**Figure 8D:**

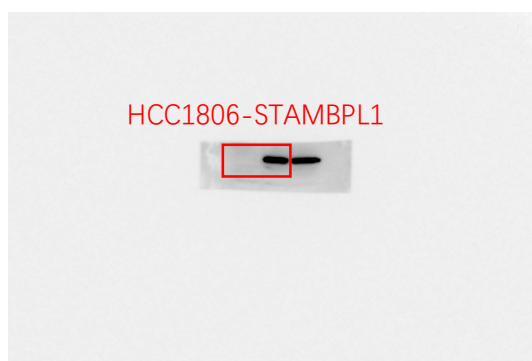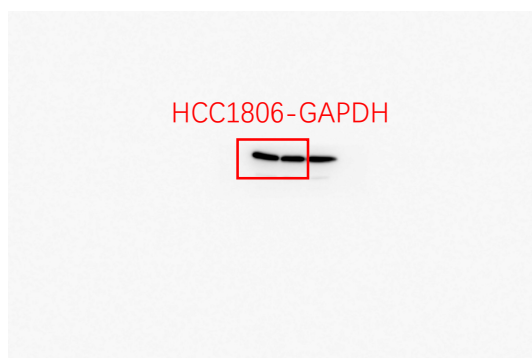

Supplement: Figure 8—source data 1. [file elife-102433-fig8-data1.zip › Figure 8-source data 1/Figure 8-source data 1.pdf]
