# Supplementary material for: STAMBPL1 activates the GRHL3/HIF1A/VEGFA axis through interaction with FOXO1 to promote angiogenesis in triple-negative breast cancer
Source: eLife. 2025 Apr 10;13:RP102433. doi: 10.7554/eLife.102433 (PMC11984952; doi:10.7554/eLife.102433)

Figure 7-figure supplement 1A:

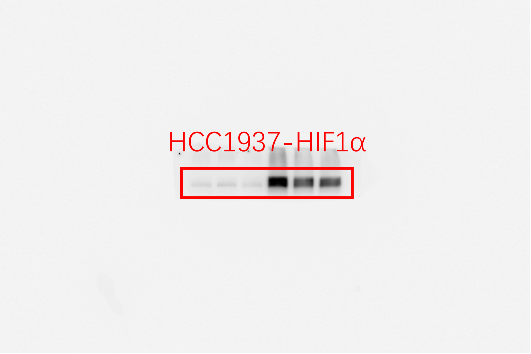

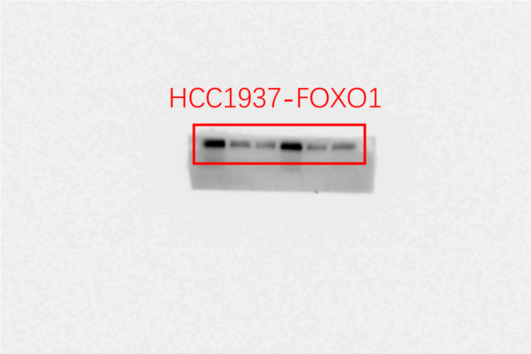

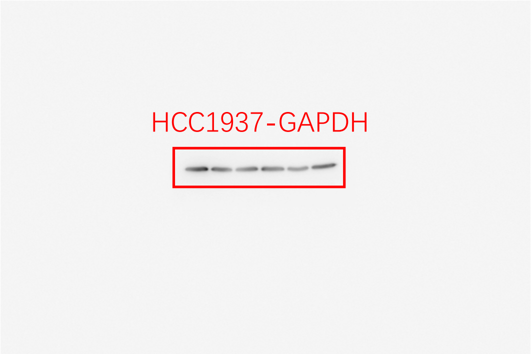

Figure 7-figure supplement 1E:

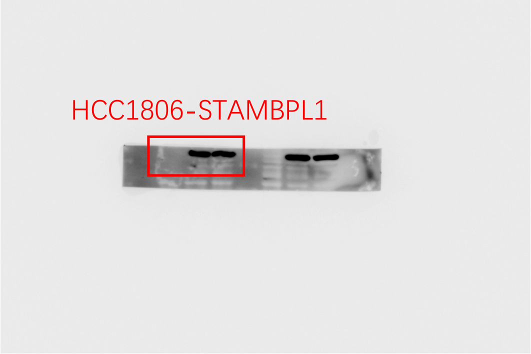

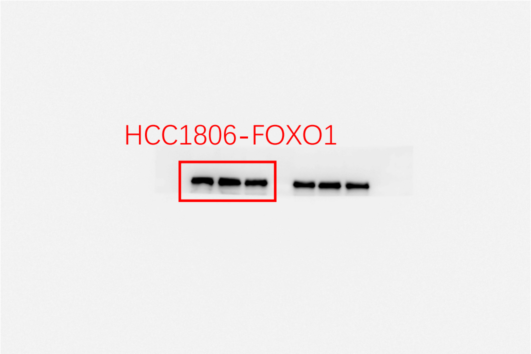

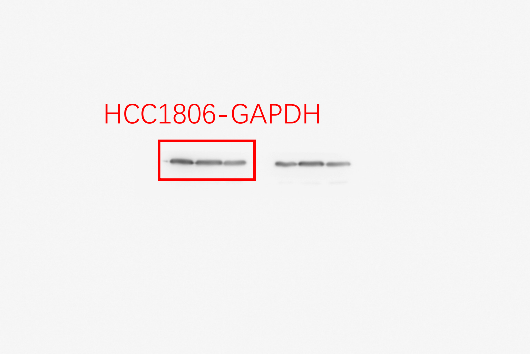

Figure 7-figure supplement 1I:

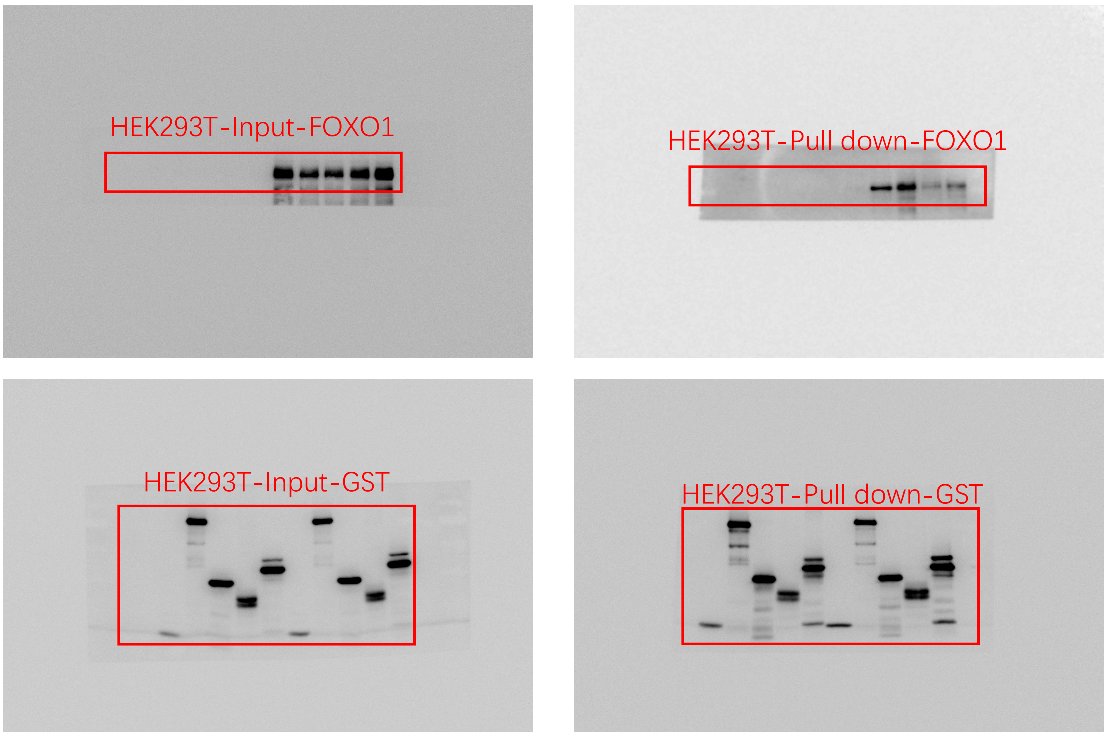

Figure 7-figure supplement 1J:

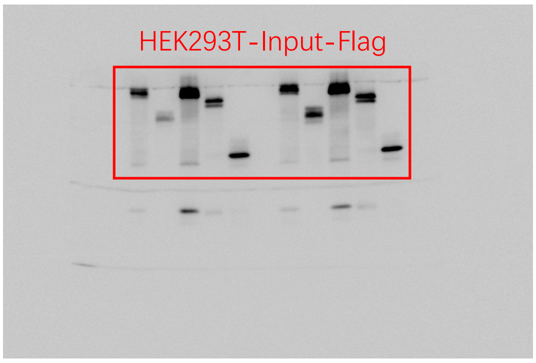

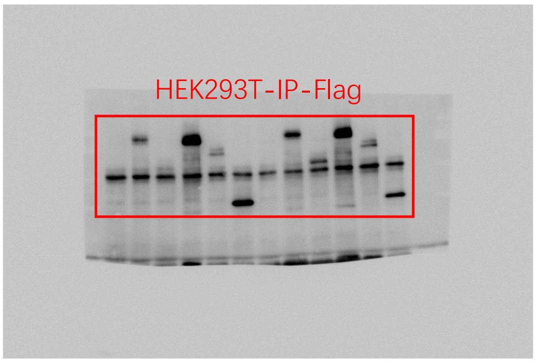

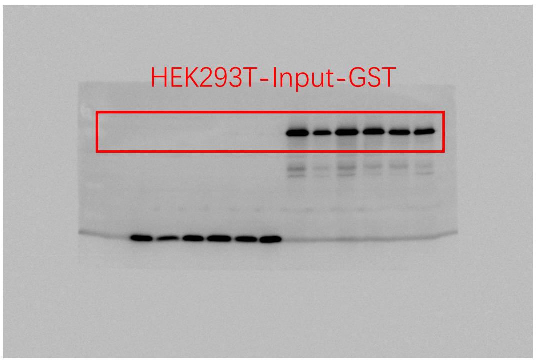

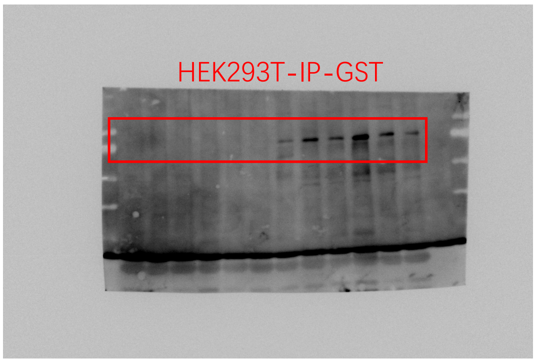

Supplement: Figure 7—figure supplement 1—source data 1. [file elife-102433-fig7-figsupp1-data1.zip › Figure 7-figure supplement 1-source data 1/Figure 7-figure supplement 1-source data 1.pdf]
